# Evaluation of Biosecurity Measures in Pig Holdings in Slovenia as a Risk Assessment for the Introduction of African Swine Fever Virus

**DOI:** 10.3390/pathogens12030434

**Published:** 2023-03-09

**Authors:** Jan Plut, Tanja Knific, Irena Golinar Oven, Gorazd Vengušt, Marina Štukelj

**Affiliations:** 1 Clinic for Ruminants and Pigs, Clinic for Reproduction and Large Animals, Veterinary Faculty, University of Ljubljana, 1000 Ljubljana, Slovenia; 2 Institute of Food Safety, Feed and Environment, Veterinary Faculty, University of Ljubljana, 1000 Ljubljana, Slovenia; 3 Institute of Pathology, Wild Animals, Fish and Bees, Veterinary Faculty, University of Ljubljana, 1000 Ljubljana, Slovenia

**Keywords:** African swine fever, biosecurity, commercial pig farms, non-commercial pig farms, outdoor pig farms, risk assessment

## Abstract

African Swine Fever (ASF) is persistently spreading and hindering pork production in Europe. Slovenia is one of the last countries in Central Europe without a confirmed ASF case in domestic pigs or in wild boar. The aim of this study was to assess the current biosecurity implementation on different types of pig farms. Internal and external biosecurity status was determined in 17 commercial (CF), 15 non-commercial (NC), and 15 outdoor (O) farms. Data were collected using the Biocheck.UGent questionnaire and assessed in combination with the latest information on the wild boar population in Slovenia. Biosecurity was compared between farm types based on the assessment of 12 subcategories. Statistically significant differences (*p* < 0.05) were found in six subcategories: (i) purchase of pigs and semen, (ii) visitors and farmworkers, (iii) vermin and bird control, (iv) finishing unit, (v) measures between compartments and use of equipment, and (vi) cleaning and disinfection. The highest total biosecurity score (0–100%) was determined on CF with 64.59 ± 16.47%, followed by NC with 55.73 ± 10.67%, and O with 48.47 ± 8.20%. The density of the wild boar population was estimated from the number of wild boars per km^2^ per year, with 3 or more hunted wild boars per unit representing the highest density. Geolocation of farms on the wild boar population map showed that two O farms are at high risk and seven farms (1 O, 5 NC, and 1 CF) are at medium risk for disease transmission from wild to domestic pigs. Biosecurity measures must be tightened in some subcategories, especially in areas with a high density of wild boar.

## 1. Introduction

African Swine Fever (ASF) is caused by a virus of the genus *Asfavirus* [1] and is considered the most important swine disease of this century [2]. The disease has been continuously spreading and hampering pig production in Europe since 2007, when the outbreak was first detected in wild boar in the Eurasian-Caucasian country of Georgia [3]. From there, ASF quickly spread throughout the Caucuses to Armenia, Azerbaijan, Abkhazia, and South Ossetia and into the wild boar population in the Chechen republic in the Russian Federation [4]. Outbreaks were detected in Ukraine in 2012 [5] and in Belarus in 2013 [6], bringing ASF to the eastern border of the European Union, which was crossed in 2014 in Poland [7], Lithuania [8,9], Latvia, and Estonia [10]. Although ASF spread inside the European Union is mostly slow, cases of long-distance disease jumps were confirmed, namely in the Czech Republic [11,12] and in Belgium [13]. New outbreaks in European wild boar and domestic pigs have been detected every year and tracked through the Animal Disease Information System (ADIS) [14]. Although most outbreaks in Europe are associated with wild boar, their population on the continent is not declining [15]. ASF has become endemic in some European wild boar populations and occurs in repeated cycles [16]. In Romania, on the other hand, 1073 ASF cases were confirmed in domestic pigs by October 2018 while only 155 cases were confirmed in wild boar [17]. ASF causes severe clinical disease in naïve pig populations, resulting in devastating direct economic losses [18]. The steady rate of spread, lack of adequate vaccine [19], and the persistence of the infectious virus in fomites and pork products [20] require international market restrictions that cripple the pig production industry and food trade [14]. Given the reservoir in the wild boar population, national and international restrictions based on strict biosecurity measures are the only prevention against the spread of ASF.

Each country that produces pork establishes its own internal laws associated with its customs and traditions that ultimately impact biosecurity; the bottom line is that biosecurity can never be perfect, certainly not for diseases with ASF-like characteristics. In order to be prepared for a possible outbreak and not just prevent it, implemented biosecurity measures must first be analyzed and evaluated. Various tools and methods can be used to evaluate biosecurity, including the use of protocols, predictive models, and biomarkers [21,22,23,24]. When using protocols or questionnaires, the broader possibility of evaluating the collected information and interpreting the results is prevented by the different characteristics of pig farms. It is also important to critically and thoroughly evaluate the link between wild and domestic pigs; in Europe, most outbreaks are reported in wild boars, which may pose a direct or indirect risk for disease transmission to domestic pigs by pig handlers [25]. Similar cases have already occurred in some European countries, e.g., in Romania (epidemiological assessment of the outbreak stated that wild boar were most likely associated with transmission to domestic pigs) [26], Bulgaria (the possibility of transmission by wild boar was considered low to moderate) [27], and Serbia (transmission by wild and domestic pigs was considered very unlikely in a particular case report) [28], to name the countries closest to Slovenia that reported outbreaks in domestic pigs. Pig farming in these areas still consists of mostly small backyard farms with almost no biosecurity measures. The situation is similar in Slovenia, where most pig farms are small-scale and only a few pigs are kept for family consumption [29]. There are no feral domestic pigs living in Slovenia, but the Slovenian hunting association reports some boar-pig hybrids in the area, which are included in the wild boar population [30]. In other neighboring countries, ASF cases in wild boar were confirmed in Hungary in 2018 [17] and in northern mainland Italy in 2021 [31].

The objective of this study was to estimate and evaluate the risk of ASF introduction into the domestic pig population based on biosecurity measures in three types of pig farms: commercial farms (CF), non-commercial farms (NC), and outdoor farms (O). The density of the wild boar population in the vicinity of the sampled farms was determined to assess the risk of transmission from one species to the other.

## 2. Materials and Methods

### 2.1. Biosecurity Assessment with BUG Questionnaire

The Biocheck.UGent (BUG) questionnaire is a free online tool for welfare assessment that can accessed through https://biocheckgent.com/en/questionnaires/pigs (accessed on 3 March 2023). Registered users are guided through a series of questions, always in the same order; the same is then projected to the answerer, who is a farm owner or production manager. The maximum number of questions is 109, plus 8 or 9 introductory questions; 78–79 questions are “Yes or No”, 11 questions are answered with a number, and 28 questions are “fill-in” type. There are 12 different indicators (variables) of biosecurity (for the sake of clarity we have given them shorter names in brackets):

- Purchase of breeding pigs, piglets, and semen (purchase)
- Transport of animals, removal of carcasses, and manure (transport)
- Supply of feed, water, and equipment (supplies)
- Visitors and workers (people)
- Pest and bird control (pests)
- Farm location (location)
- Disease management (disease management)
- Farrowing and farrowing period (farrowing and suckling)
- Nursery unit (nursery)
- Finishing unit (finishing)
- Measures between units, work lines, and equipment use (biosecurity between units)
- Cleaning and disinfection (disinfection)

BUG questionnaires were completed during farm inspections in 2021 and 2022 as part of the two-year Slovenian Target Research Program (Slovenian acronym: CRP), called CRP V4-2039, entitled risk-assessment of the introduction of African swine fever virus into domestic pig holdings (between 2021 and 2023). Responses were then entered into an online tool that calculated biosecurity indicators. Based on the representative number of questionnaires received, the average biosecurity rating in Slovenia was calculated. A detailed summary was prepared for each individual farm, including a comparison with Slovenia and the world as well as general biosecurity recommendations. We then exported the data and calculated results from the online platform and made calculations of basic descriptive statistics using R software version 4.2.1 [32] for all farms and each category of farm: commercial (*n* = 17), non-commercial (*n* = 15), and outdoor (*n* = 15). Differences in biosecurity assessments between breeding categories were determined based on the number of groups in the case of normal distribution of data using the parametric ANOVA test. In the event that the assumption of normal distribution was not satisfied, the non-parametric Wilcoxon or Kruskal-Wallis rank sum test was used. For all statistical tests, the threshold for statistical significance was a *p* value lower than 0.05.

### 2.2. Wild boar Population Estimation

Based on the data submitted by hunters and hunter families throughout Slovenia, a risk assessment was conducted and a heat map for the transmission of wild and domestic pigs was created. The density of wild boar was determined by the number of animals shot per square kilometer over a 5-year period (2017–2022) and was divided as follows:

- More than 3 wild boar/km^2^; highest risk
- 2–3 wild boar/km^2^
- 1–2 wild boar/km^2^
- 0.5–1 wild boar/km^2^
- 0–0.5 wild boar/km^2^; lowest risk

## 3. Results

### 3.1. BUG Questionnaire and Farm Comparisson According to the Farm Type

Based on the answers in the pre-prepared BUG questionnaire, biosafety indicators were calculated, which were then combined into an assessment of internal and external biosafety and a total assessment. Every question from the BUG questionnaire was assessed and compared based on the farm type represented. Out of the 9 introductory questions, the answers differentiated significantly for 7 questions. The other 109 questions belonged to 12 subcategories, among which the answers to 41 questions differentiated specifically depending on the type of farm investigated. Statistically significant differences (*p* < 0.05) were found in 6 out of 12 subcategories for biosecurity evaluation: (i) purchase of pigs and semen, (ii) visitors and farmworkers, (iii) vermin and bird control, (iv) finishing unit, (v) measures between compartments and use of equipment, and (vi) cleaning and disinfection. Based on these subcategories, statistically significant differences were also determined for main categories, i.e., the internal, external, and total biosecurity scores. The results with statistically significant differences are shown in Table 1. In all cases, the results showing statistically significant differences were present at outdoor farms compared to the other two types of farms.

**Figure 1:**
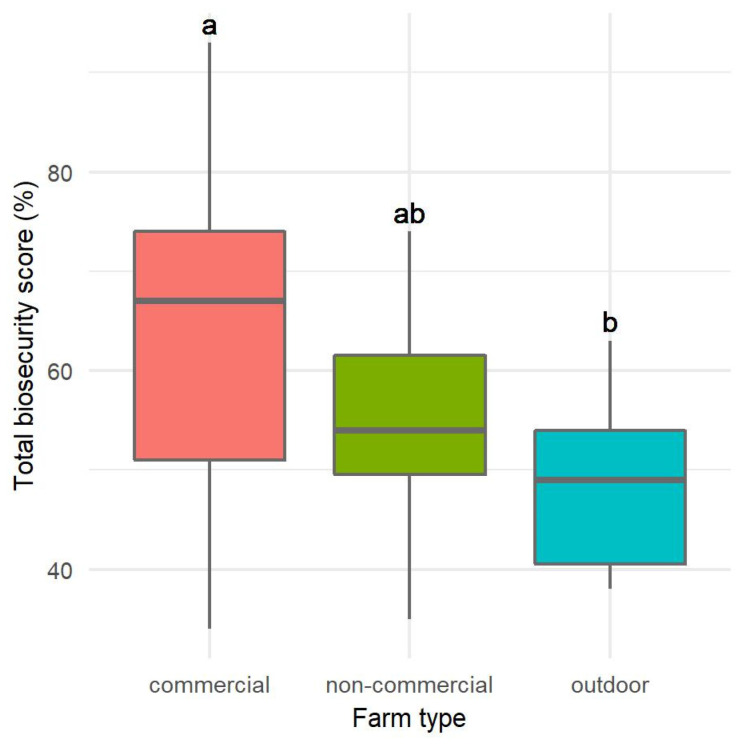
Distribution of the total biosecurity assessment scores according to the type of farm. Statistically significant differences (*p* < 0.05) observed between farm types are marked with different lower-case letters.

Commercial and outdoor farms differed statistically in the total biosecurity assessment (Kruskal-Wallis test, *p* = 0.0093), while there were no differences between the other pairs. On average, CF had the highest total biosecurity score of 64.59 ± 16.47% (mean ± standard deviation), NC had a significantly lower score (55.73 ± 10.67%), and O had the lowest score with an average of 48.47 ± 8.20%. In Figure 1, we show the distribution of the data using frames with handles, where the median (thick line in the frame) and first and third quartiles are visible, which show half of all data (frames), the minimum and maximum (handles), respectively, and the conditional minimum and maximum, if outliers are present.

**Figure 2:**
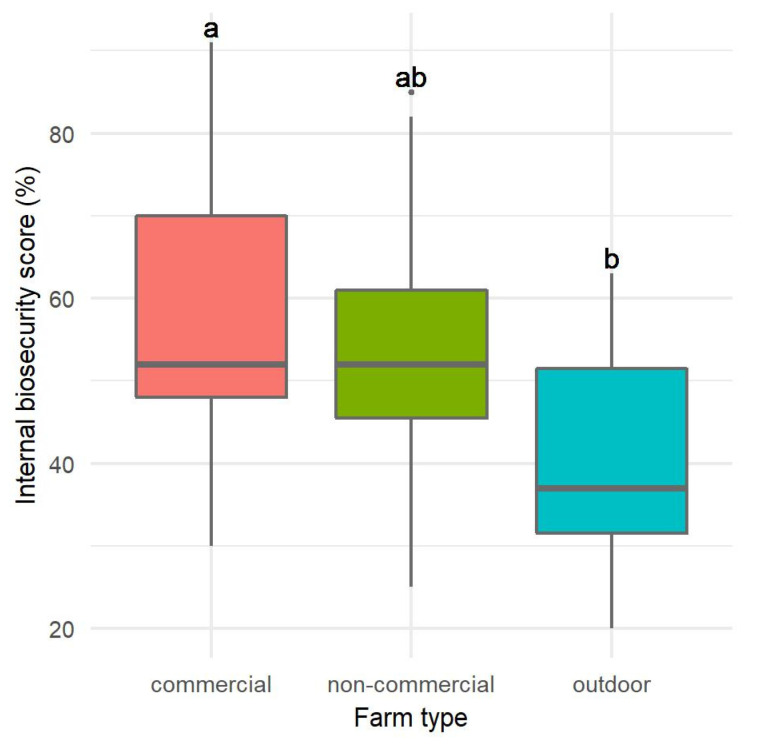
Distribution of the assessment of internal biosecurity scores according to farm type. Statistically significant differences (*p* < 0.05) observed between farm types are marked with different lower-case letters.

**Figure 3:**
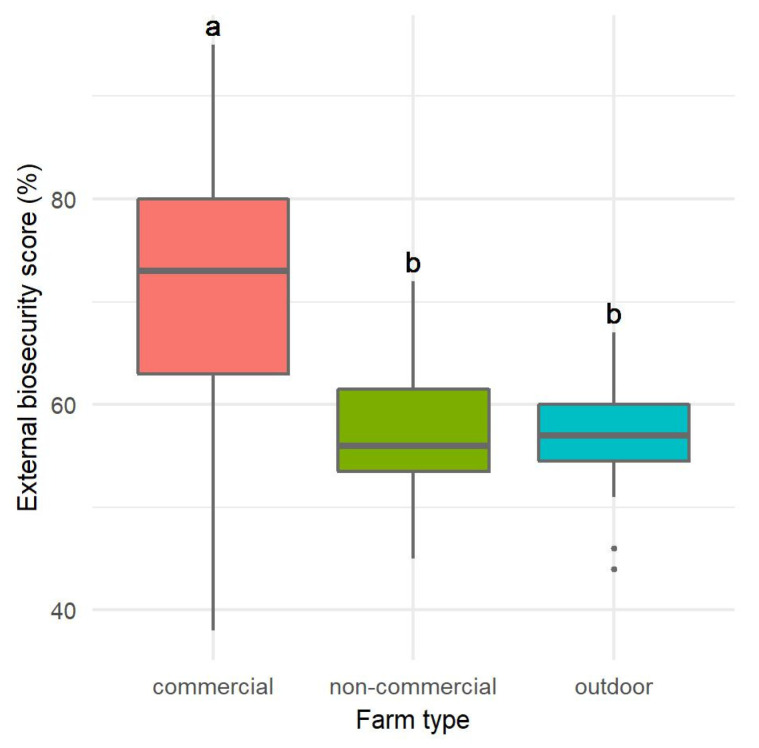
Distribution of the assessment of external biosecurity scores according to farm type. Statistically significant differences (*p* < 0.05) observed between farm types are marked with different lower-case letters.

The assessment of internal biosecurity (Figure 2) was also statistically different only between CF and O (ANOVA, *p* = 0.0069); it was also highest in CF (59.12 ± 18.20%), slightly lower in NC (54.13 ± 16.39%), and lowest in O (40.40 ± 13.43%). The assessment of external biosecurity in CF was statistically significantly different from the assessment in NC and O (Kruskal-Wallis test, *p* = 0.0099), namely it was highest in CF (69.65 ± 16.81%), and lower but comparable in NC and O (57.00 ± 6.71% and 56.13 ± 5.89%, respectively) (Figure 3).

According to the individual biosecurity indicators, the largest differences were between CF and O. There were statistically significant differences in the indicators of people (Kruskal-Wallis test, *p* < 0.0001), pests (ANOVA, *p* = 0.0006), biosecurity between units (Kruskal-Wallis test, *p* = 0.0013), and cleaning (Kruskal-Wallis test, *p* = 0.0003). In the indicators of people and pests, commercial and non-commercial farms also differed statistically, while the difference between NC and O was not statistically significant. In cleaning, O also differed statistically significantly from NC. For the finishing pigs NC was statistically significantly different from CF and O (Kruskal-Wallis test, *p* = 0.0022), while for the purchase indicator, the difference was statistically significant only between NC and O (Kruskal-Wallis test, *p* = 0.0114).

Individual indicators for which the husbandry categories did not statistically differ were: transport (Kruskal-Wallis test, *p* = 0.1495), stocks (ANOVA, *p* = 0.1580), location (ANOVA, *p* = 0.1820), disease management (Kruskal-Wallis test, *p* = 0.0697), farrowing and suckling (Wilcoxon test, *p* = 0.6465), and nursery (Wilcoxon test, *p* = 0.1318). A summary of data by individual indicators is presented in Table 2.

### 3.2. Risk of Disease Transmission to Farms from Wild Boars in Slovenia

**Figure 4:**
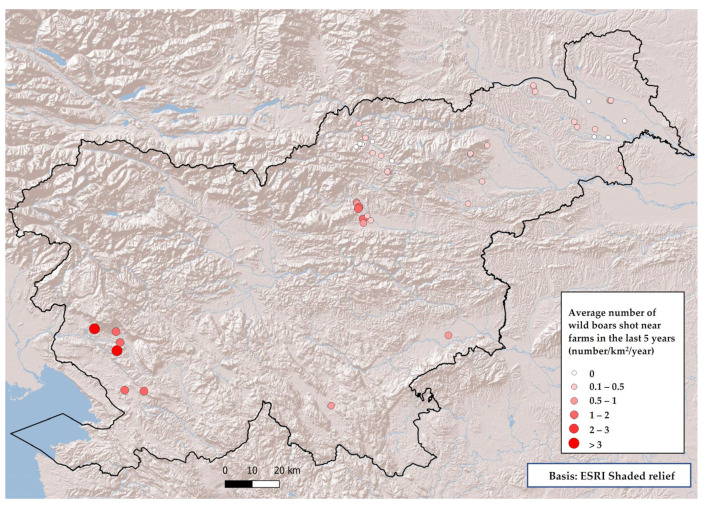
Mapped locations of the farms marked with circles representing the number of shot wild boar per km^2^ in the area.

Data provided by hunters were collected and analyzed and a heat map was created for risk assessment, in which farms were plotted. A total of 9 farms from the study were classified as high or medium risk if the number of wild boar shot in the area was more than 3 or at least 1.5-2 wild boar shot per km^2^ (Table 3, Figure 4).

## 4. Discussion

The data collected with the BUG questionnaire revealed some general information about biosecurity in Slovenian pig farms and their deficiencies and shortcomings. This was the first real assessment of biosecurity in this country, and the results of the collected data confirmed our prediction that biosecurity measures in general could and should be better. A superficial comparison of Slovenian averages with the global average showed that pig farms underperformed on 11 out of 12 observed variables. To be fair, for the national and world average data to be more meaningful, we would need to evaluate the details on the types of establishments that provided responses to the BUG questionnaire database. We collected data relatively evenly for each given farm type, while this may not be the case in other countries. A comparison of results between farm types also confirmed our assumption that biosecurity was significantly lower in NC and O farms than in CF, which was consistent with the results of other similar studies comparing biosecurity in different pig farm types [33,34,35]. In terms of external biosecurity on outdoor farms, which represents the most obvious route of transmission from wild to domestic pigs, statistically significant differences were found between outdoor farms and the two other farm types for the purchase of breeding pigs, piglets, and semen; visitors and employees; and pest and bird control. These risks could be addressed first when considering how to improve biosecurity measures. First, outdoor farms should be properly (double) fenced to prevent direct contact between livestock, feral pigs, and wild boars [35]. Especially for farms located in areas with a high density of wild boar, such as the two farms in southwestern Slovenia, this should be a necessity. Some outdoor farm problems are difficult to remedy, i.e., it is impossible to prevent contact between pigs and other animal species, even vermin such as rodents and birds. However, the number of visitors could be reduced to a minimum by educating everyone on site about the importance of putting on clean clothes and boots, paying attention to general and hand hygiene, keeping a visitors’ book, etc.

European and Slovenian laws state that once an ASF case is confirmed in domestic pigs, all pigs in a 3 km diameter must be destroyed [36,37], which would virtually wipe out small-scale pig production in infected areas. In Slovenia, a farm with about 50 breeding pigs is considered quite large and can provide a source of live animals or pork for a respectable portion of the surrounding region or neighborhood. To put this statement into perspective: the Republic of Slovenia Statistical Office stated that all agricultural holdings bred a total of approximately 216,000 pigs in 2021, and that was the lowest number since 1991 [38]. A report from the Biotechnical Faculty (Department of Animal Science) from 2021 stated that there were 12,903 pig farms in Slovenia, of which 9,931 (77%) farms only bred fatteners. There were large farms that only bred fatteners (more than 200 fatteners), only two farms (0.07%) had more than 1000 breeding sows, and 2646 farms (93.8 %) with breeding sows had less than 25 sows. More than 95% of all pigs are in the eastern part of the country [29]. Pig production in Slovenia is already one of the smallest in Europe, and the introduction of ASF could be the final blow to an already struggling sector. Something needs to be done, and a consensus between authorities and producers would be the ideal solution. A silver lining, if there is one, is the fact that NC and O farms are separated from the few CF still operating in Slovenia. However, even if most swine farms in the country are kept free of ASF, restrictions at all levels would impact trade and make it difficult to move live pigs and products. Enforcement of some biosecurity measures is and will be necessary.

Fortunately, Slovenia has remained ASF-free up to 2023; unfortunately, even the pessimistic estimate of the Slovenia Forest Service predicts that the wild boar population will grow in the next decades. However, the results and analyses from our study should help make it slightly easier to prepare for a potential outbreak of ASF in Slovenia, which is highly likely in the wild boar population, but we think it can be prevented in domestic pig farms. The annual national order on the implementation of systematic monitoring of animal health, animal disease eradication programs, and animal vaccinations in 2021 was the first to oblige field veterinary practitioners to routinely assess biosecurity on pig farms [39]. An official national regulation on biosecurity is about to be issued in the near future and data collected by the annual order will serve for the official categorization of farms. Regulations on biosecurity will set a mandatory rule for farms to have an internal biosecurity plan. Since we now have black and white information about the biggest holes in biosecurity, especially regarding external biosecurity on outdoor farms (with purchase, visitor, and vermin being the most critical biosecurity risks for outdoor farms), hopefully these results will be used by institutions to implement laws and try to find solutions for farmers and pig production. Hopefully, new regulations on biosecurity will be implemented soon.

## 5. Conclusions

Our results confirmed that biosecurity measures on pig farms are significantly less strict on outdoor pig farms, which was expected considering the results of some previous studies. Through an analysis of critical biosecurity variables, we managed to present the biggest biosecurity flaws. An analysis of the wild boar population predicts further growth of the population, thus increasing the transmission risk from wild to domestic pigs. Hopefully, our results will be helpful when implementing new national regulations on biosecurity and will contribute to a high-quality legal act.

## Figures and Tables

**Table 1:** Subcategories and total biosecurity score and statistically significant differences between farm types. Results are based on the BUG questionnaire answered by farm owners.

Variable	Test	*p*-Value	Difference between Farm Types (CF:NC:O)
purchase	Kruskal-Wallis	0.0114 *	NC:O
transport	Kruskal-Wallis	0.1495	
supply	ANOVA	0.1580	
people	Kruskal-Wallis	<0.0001 *	CF:NC and NC:O
vermin	ANOVA	0.0006*	CF:NC and CF:O
location	ANOVA	0.1820	
disease	Kruskal-Wallis	0.0697	
farrowing and suckling	Wilcoxon	0.6465	
nursery	Wilcoxon	0.1318	
finishing	Kruskal-Wallis	0.0022 *	CF:NC and NC:O
measures	Kruskal-Wallis	0.0013*	CF:O
disinfection	Kruskal-Wallis	0.0003 *	CF:O and NC:O
**external biosecurity**	Kruskal-Wallis	0.0099 *	CF:NC and CF:O
**internal biosecurity**	ANOVA	0.0069 *	CF:O
**total biosecurity score**	Kruskal-Wallis	0.0093 *	CF:O

* statistically significant difference between at least two types of farms, CF–commercial farms, NC–non-commercial farms, O–outdoor farms.

**Table 2:** Summary variables by farm type and total for all Slovenian farms (average ± standard deviation in percentage).

Variable	Farm Type			Total for Slovenian Farms	World Average
	CF	NC	O		
purchase	87.06 ± 13.89	98.13 ± 4.98	85.33 ± 15.02	90.04 ± 13.24	88
transport	70.53 ± 20.59	61.20 ± 15.33	60.47 ± 17.68	64.34 ± 18.34	80
supply	49.06 ± 19.40	38.93 ± 16.68	40.00 ± 10.72	42.94 ± 16.50	49
people	70.59 ± 31.97	18.27 ± 10.76	18.60 ± 10.05	37.30 ± 32.61	73
vermin	71.76 ± 21.28	39.33 ± 26.04	50.00 ± 19.27	54.47 ± 25.86	77
location	51.76 ± 30.67	55.33 ± 19.95	67.33 ± 19.07	57.87 ± 24.58	67
disease	57.65 ± 35.97	28.00 ± 34.48	40.00 ± 26.19	42.55 ± 34.29	76
farrowing and suckling	67.35 ± 13.54	/	68.80 ± 17.68	67.89 ± 14.88	61
nursery	64.29 ± 22.67	/	51.50 ± 18.52	59.56 ± 21.78	68
finishing	50.50 ± 25.28	79.60 ± 14.83	53.07 ± 26.57	61.30 ± 25.95	78
measures	55.71 ± 21.72	45.00 ± 18.44	34.73 ± 11.61	45.60 ± 19.62	56
disinfection	58.35 ± 30.81	62.00 ± 20.94	18.67 ± 25.60	46.85 ± 32.34	72

CF–commercial farms, NC–non-commercial farms, O–outdoor farms

**Table 3:** Average number of shot wild boar (wild boar/km^2^/year) and farm locations (statistical region). Nine farms with high or medium risk are sorted from highest to lowest.

Farm Type	Statistical Region	Average No. of Boar Shot/Km^2^/Year	Risk
O	Gorizia (southwest)	3.42	high
O	Coastal–Karst (southwest)	3.04	high
NC	Savinja (central-east)	1.96	medium
O	Gorizia (southwest)	1.87	medium
NC	Savinja (central-east)	1.80	medium
NC	Coastal–Karst (southwest)	1.60	medium
NC	Coastal–Karst (southwest)	1.58	medium
NC	Gorizia (southwest)	1.51	medium
CF	Savinja (central-east)	1.27	medium

Farms in the area with less than 1 wild boar shot/km^2^/year are not shown in the table, CF–commercial farms, NC–non-commercial farms, O–outdoor farms.

## Data Availability

All original data in this study are available from the authors of the study upon request. Publicly available portals are listed in the Reference section.
